# Intracranial electrophysiological biomarkers of compulsivity in obsessive–compulsive disorder

**DOI:** 10.1038/s44220-025-00457-9

**Published:** 2025-07-24

**Authors:** Tara Arbab, Melisse N. Bais, Martijn Figee, Isidoor O. Bergfeld, P. Richard Schuurman, Pepijn van den Munckhof, Ingo Willuhn, Damiaan Denys

**Affiliations:** 1 Department of Psychiatry, Amsterdam UMC, University of Amsterdam, Amsterdam, The Netherlands; 2 Amsterdam Neuroscience, Amsterdam, The Netherlands; 3 Amsterdam Brain and Cognition, University of Amsterdam, Amsterdam, The Netherlands; 4 Netherlands Institute for Neuroscience, Royal Netherlands Academy of Arts and Sciences, Amsterdam, The Netherlands; 5 Nash Family Center for Advanced Circuit Therapeutics, Icahn School of Medicine at Mount Sinai, New York, NY, USA; 6 Department of Neurosurgery, Amsterdam UMC, University of Amsterdam, Amsterdam Neuroscience, Amsterdam, The Netherlands

## Abstract

There is an emerging need for objective neural biomarkers of obsessive–compulsive disorder (OCD) to improve the efficacy of neuromodulatory interventions, most notably deep-brain stimulation (DBS), and develop closed-loop stimulation paradigms. Preliminary data suggest that such biomarkers may be derived from local field potentials (LFPs) recorded in individual patients implanted with sensing DBS devices. However, reliable LFP signatures that are generalizable across OCD patients have yet to be identified. Here, we relate LFPs recorded from sensing DBS electrodes in different basal-ganglia structures to core symptoms of OCD in 11 patients during personalized provocation of obsessions and compulsions. We identify two general markers of compulsion: delta and alpha LFP power was significantly increased during all compulsions in the external globus pallidus (GPe), nucleus accumbens, anterior limb of the internal capsule (ALIC) and anterior lateral anterior commissure. In mental compulsion subtypes, similar low-frequency increases were observed only in GPe (delta/alpha) and ALIC (alpha), suggesting that these signals possibly reflect more universal biomarkers of compulsivity unconfounded by motor function. GPe delta power correlated with OCD symptom severity, establishing a meaningful connection between subcortical sensing DBS readout and patient experience. ALIC alpha power was modulated by the phase of theta oscillations during compulsions, possibly reflecting pathological coupling of cortical networks in OCD. Our results demonstrate unique, group-level LFP correlates of core OCD symptoms across disease-relevant basal-ganglia structures. These electrophysiological signatures help pave the way toward the development of biomarker-targeted neuro-modulatory intervention for OCD. Netherlands Trial Register ID: NL7486.

Obsessive–compulsive disorder (OCD) is a debilitating psychiatric disorder characterized by persistent, unwanted intrusive thoughts (obsessions) and corresponding repetitive, ritualistic behaviors (compulsions). Although the majority of patients can be treated to a satisfactory level with pharmacotherapy and behavioral therapy, approximately 10% of patients respond insufficiently and remain severely ill^1^. In these therapyresistant patients, neuromodulation using deep-brain stimulation (DBS) can be highly effective, leading to response rates of 52–66% (refs. 2,3).

DBS entails neurosurgical implantation of intracranial electrodes to target specific brain areas with electrical pulses, modulating activity of local and connected neuronal networks. In therapy-resistant OCD, common DBS targets are the internal capsule, the nucleus accumbens (NAc) and the subthalamic nucleus^4,5^. These structures relate to the cortico-striato-thalamo-cortical (CSTC) circuit, in which aberrant neuronal network activity is hypothesized to underlie OCD^6–11^. However, the exact physiological manifestation of OCD in CSTC structures is yet to be discovered, and approximately 30–50% of patients experience partial or inadequate response to DBS, remaining severely disabled in daily functioning^2^.

Improved understanding of electrophysiological activity throughout CSTC networks could improve clinical outcomes of neuromodulatory interventions such as DBS^12–14^. Novel technological advances allow real-time recording of intracranial brain activity (neuronal oscillations), time-locked to behavioral states, directly from DBS electrodes, from which neural correlates of OCD may be obtained. These oscillations, derived from the local field potential (LFP), are extracellular electrical signals that reflect temporal coordination of neuronal activity^15^. The frequency and amplitude of this synchronized neuronal network activity has been functionally linked with cognitive and mechanistic processes throughout the brain, such as neuronal communication and information processing^16,17^; furthermore, abnormal LFP oscillations have been linked with pathological neuronal activity in modeled psychiatric disease^18^. As altered communication between cortical- and basal-ganglia structures is thought to underlie OCD^19^, we hypothesized that OCD symptoms would be reflected as compromised LFP in these networks. Indeed, case reports^20–24^ characterizing neural correlates of OCD provide insight into possible neuronal signatures on the level of individual patients, and, in a recent publication from our team, deep-learning models based on LFP recordings from individual patients could identify different symptom states whereas models based on (group-level) data from multiple patients could not^25^. However, targeted intervention for OCD symptoms (using neuromodulation such as so-called closed-loop DBS, where stimulation adapts directly to intervene with detected pathological neural activity) requires general, group-level disease biomarkers.

Here, we present LFP data from 11 sensing-DBS-implanted OCD patients, recorded in basal-ganglia nuclei and associated white matter, aiming to discover electrophysiological correlates related to cardinal features of OCD across patients and brain regions. We employed a behavioral task that effectively evoked core symptoms, imitating real-life brain states in which patients might benefit from improved neuromodulatory intervention, and identify general disease biomarkers throughout the basal ganglia and associated white matter.

## Results

**Fig. 1:**
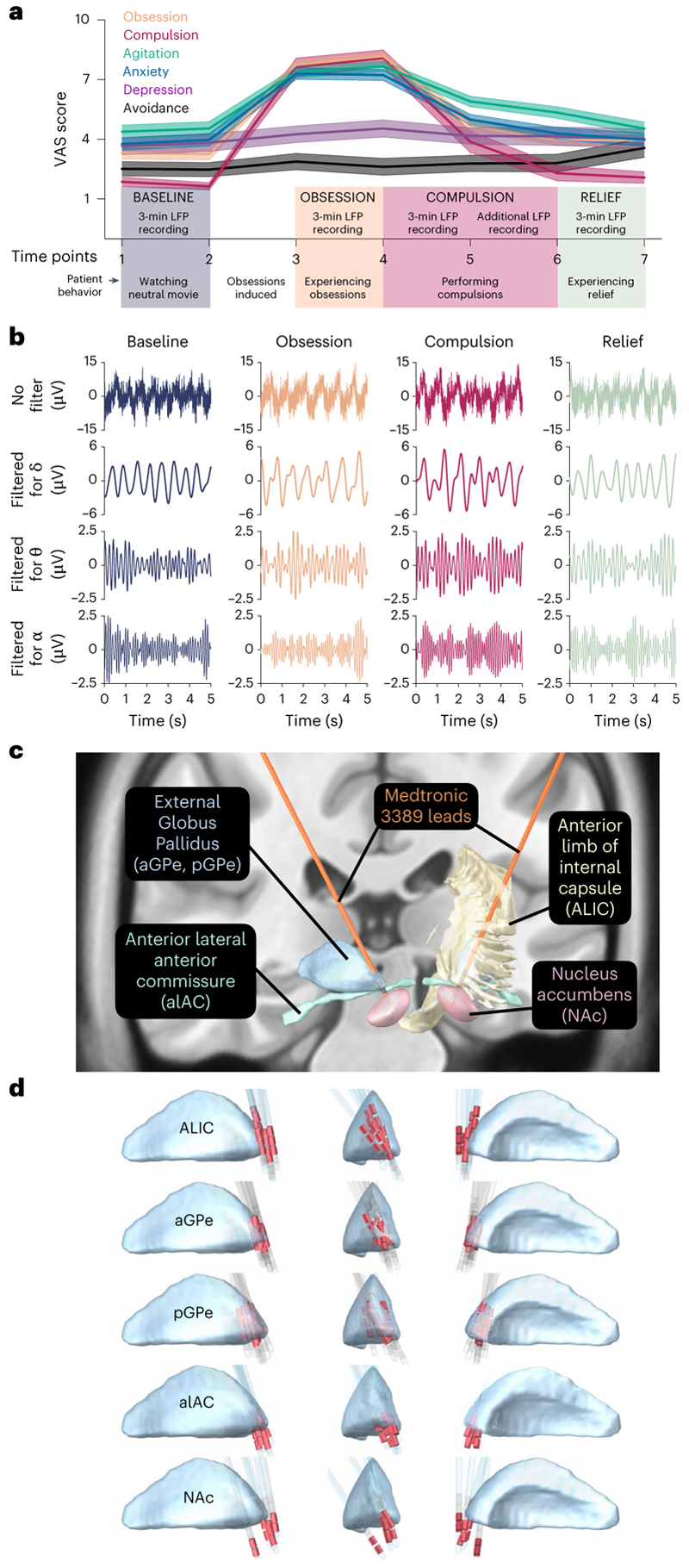
Intracranial electrophysiological recordings during symptom provocation in OCD. **a**, OCD symptoms were successfully induced by our provocation paradigm. Patients (*n* = 11) reported VAS scores reflecting the experience severity of obsession, compulsion, anxiety, agitation, depression and avoidance at seven timepoints throughout symptom provocation (mean ± s.e.m.). Three minutes of LFP was recorded during each behavioral condition: during ‘baseline’ patients watched a neutral movie while seated in a chair; during ‘obsession’ and ‘compulsion’ they respectively experienced and performed their patient-specific obsessions and compulsions; during ‘relief’ they were again seated in a chair. Additional compulsion LFP was recorded if the patient could not cease this behavior after 3 min. **b**, Example LFP traces recorded during symptom provocation. Top rows show full-spectrum LFP, bottom rows show the same trace filtered for different frequency bands. **c**, Anatomical localization of the DBS leads (Medtronic 3389, orange) from which LFP was recorded in one example patient, illustrating in coronal view the basal-ganglia nuclei (GPe, NAc) and associated white-matter structures (ALIC (shown only unilaterally), alAC) that were targeted in this study. **d**, Highlighted (in red) Medtronic 3389 contact electrodes from which LFP was recorded in this study projected onto one hemisphere, separated per anatomical target, shown in reference to GPe (blue structure; in lateral, frontal and medial views).

Eleven patients with treatment-resistant OCD indicated for DBS were included in this study over a period of 5 yr. Patient demographics are summarized in the left-hand panel of Table 1. All patients were implanted bilaterally in the brain with a Medtronic DBS system and leads. The experiment took place after recovery from surgery. After a 3-min baseline (BAS) in which patients watched a neutral movie, during which LFP was recorded from the DBS electrodes, patient-specific obsessions (OBS) were provoked and LFP was recorded for 3 min in this obsessive state. Subsequently, patients performed their compulsions (COM), while LFP was recorded for at least 3 min, until patients reported their urge had subsided. Finally, 3 min of LFP was recorded as patients experienced relief (REL). Severity of obsession, compulsion, agitation, anxiety, depressive mood and avoidance was quantified throughout the experiment by patient-reported visual analog scale (VAS) scores (Fig. 1a). Provocation of obsessions sharply increased severity of obsession, compulsion, agitation and anxiety compared with baseline, as reflected in the VAS scores in Fig. 1a. This severity remained stable throughout the obsession recordings, and decreased during compulsions, returning to baseline levels during relief recordings. Thus, the symptom provocation was successful in inducing the intended effect. Patient-specific obsessions and compulsions are summarized in the right-hand columns of Table 1.

Representative LFP traces recorded during each of the four behavior states in Fig. 1b illustrate reliable neural signal with the presence of delta, theta and alpha oscillations. Lead implants were localized for each individual patient in basal ganglia and associated white matter illustrated in Fig. 1c. We included only data recorded from electrode pairs that could be unambiguously localized in the anterior limb of the internal capsule (ALIC, *n* = 10), the most anterior portion of the external globus pallidus (aGPe, *n* = 12), a slightly more posterior part of the anterior external globus pallidus (pGPe, *n* = 17), the most anterior portion of the lateral anterior commissure (alAC, *n* = 6) or the NAc (*n* = 5) (Fig. 1d); the remaining (*n* = 16) electrode pairs were excluded from analysis as their placement fell wholly or partially across anatomical borders.

**Fig. 2:**
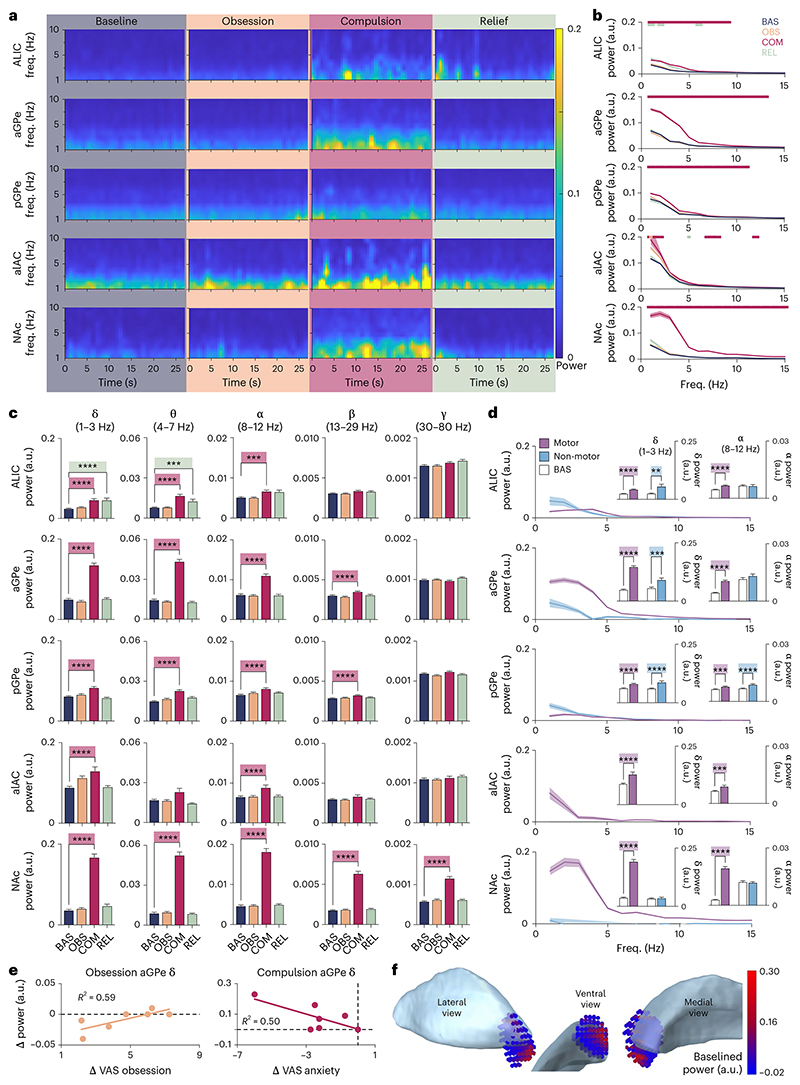
Neuronal correlates of OCD. **a**, Averaged time–frequency power spectra of the 27-s continuously recorded LFP segments in each brain region, during the experimental states. Low-frequency oscillations increased in all brain regions during compulsions. **b**, Power spectra (mean ± s.e.m.) of LFP recorded during the experimental states in each brain region. Colored, horizontal lines at the top of the graphs mark the frequencies at which the spectral power of obsession, compulsion and relief LFP significantly differed from baseline (two-tailed non-parametric randomization test, *P* < 0.05). **c**, Bar graphs (mean + s.e.m.) of spectral LFP power per frequency band during the experimental states in each brain region. Pairwise comparisons mark power during obsession, compulsion and relief significantly different from baseline (Bonferroni-corrected one-way ANOVA with Dunnett’s multiple-comparison test). Delta and alpha powers are increased during compulsions in all brain regions. **d**, Baseline-subtracted power spectra (mean ± s.e.m.) of LFP recorded during motor and non-motor compulsions in each brain region. Pairwise comparisons in bar graph insets (mean + s.e.m.) mark significantly different delta and alpha powers from baseline during (non-)motor compulsions (Bonferroni-corrected two-tailed unpaired *t*-test). **e**, Correlation and linear regression between aGPe delta power and VAS scores for obsession and anxiety during obsession and compulsion conditions. aGPe delta power significantly increased with experience of obsession during the induction and experience of obsessions (Pearson *r*(5) = .77, *P* = 0.044), whereas aGPe delta power decreased (non-significant) with reduced anxiety on performing compulsions (Pearson *r*(5) = –0.70, *P* = 0.078). **f**, Anatomical distribution of baselined delta power during compulsions across all recording sites mirrored onto one hemisphere, shown with reference to GPe (in lateral, ventral and medial views), illustrating highest power localized in NAc, ALIC and aGPe. ****P < 0.0001, ***P < 0.001, **P < 0.005. a.u., arbitrary units.

Time–frequency representation of continuous LFP recordings averaged across all patients during each behavior state demonstrates that the baseline power of low-frequency oscillations differed between anatomical recording locations but increased in all brain regions during performance of compulsions (Fig. 2a). This compulsion-driven LFP power increased significantly from baseline across multiple frequencies in all brain regions (Fig. 2b, non-parametric randomization test *P* < 0.05; Extended Data Table 1), extending heterogeneously over multiple frequency bands (Fig. 2c, Bonferroni-corrected one-way analysis of variance (ANOVA) with Dunnett’s multiple-comparison test; Extended Data Table 2). Thus, compulsions in OCD were marked by generally increased delta and alpha LFP power throughout all structures evaluated in our study, whereas increased theta, beta and gamma LFP power during compulsions was regionally distinct. Low-frequency LFP power was also significantly increased in ALIC during post-compulsion relief (Fig. 2b,c), though we cannot exclude that this was confounded by mental compulsions persisting at the start of the relief phase in one patient (which can be seen in Fig. 2a, top right panel).

As increased delta/alpha power during compulsions was observed in all brain regions, we further determined whether these signals could serve as universal compulsivity markers across the CSTC circuit. First, considering that the basal ganglia facilitate movement, we discerned to what extent our LFP signals reflected the motor component of compulsions, by dividing the data set to compare powers of delta and alpha oscillations in patients with compulsions requiring movement (for example, hand washing) and patients with non-motor/mental compulsions (for example, praying). Increased delta power during non-motor/mental compulsions persisted only in ALIC and GPe, indicating that these signals may universally mediate compulsive feelings, thoughts and actions (Fig. 2d, Bonferroni-corrected unpaired *t*-test; Extended Data Table 3); however, this signal did not persist in NAc, indicating here it may mediate only the motor-action component of compulsions, or reflect general movement. Non-motor compulsive alpha power was only increased in pGPe (Fig. 2d, Bonferroni-corrected unpaired *t*-test; Extended Data Table 4). The compulsions of all patients in the alAC group required action, so although motor-compulsion signals significantly differed from baseline in both delta and alpha bands (Fig. 2d, Bonferroni-corrected unpaired *t*-test; Extended Data Tables 3 and 4), we could not determine to what extent these signals reflect compulsive feeling.

Next, we explored the correlation between delta/alpha power and self-reported severity of obsessions, compulsions, agitation, anxiety, depressive mood and avoidance during symptom provocation in ALIC, aGPe and pGPe (that is, the regions mediating compulsive feeling). During obsessions the aGPe delta-power increase correlated positively with obsession severity (Fig. 1a: [LFP_Obsession_ – LFP_Baseline_], [VAS_Obsession(Time-point4)_ – VAS_Obsession(Time-point2)_]; Fig. 2e: Pearson *r*(5) = 0.77, 95% confidence interval 0.03093 to 0.9634, *P* = 0.044), while during compulsions the aGPe delta-power increase showed a non-significant negative correlation with anxiety severity (Fig. 1a: [LFP_Compulsion_ – LFP_Obsession_], [VAS_Anxiety(Time-point6)_ – VAS_Anxiety(Time-point4)_]; Fig. 2e: Pearson *r*(5) = –0.70, 95% confidence interval –0.9522 to 0.1053, *P* = 0.078). We found no significant symptom-severity correlations with LFP power in pGPe (delta/alpha) or ALIC (delta).

Finally, despite it being general throughout the brain regions evaluated in our study, compulsion-mediated delta-power increase showed a defined spatial distribution across all recording sites (Fig. 2f), with highest baseline-subtracted power advancing non-uniformly from NAc and ALIC into GPe. This further corroborates delta-power increase as anatomically specific (rather than general, volume-conducted) and consequential to OCD symptomatology.

**Fig. 3:**
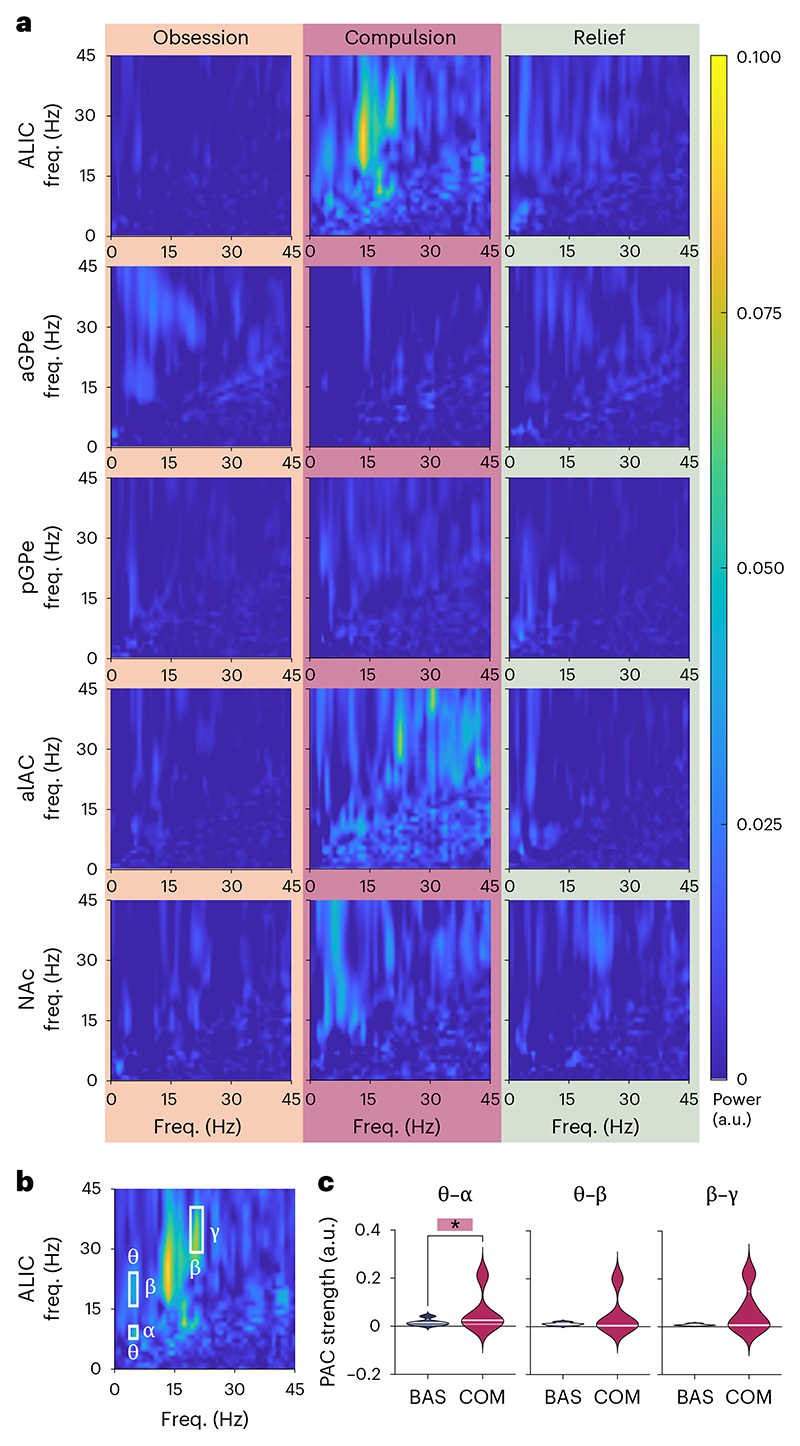
Phase–amplitude interactions. **a**, Cross-frequency coupling of baseline-subtracted LFP recorded during obsession, compulsion and relief states, averaged across patients. **b**, The highlighted areas mark increased coherence between frequency bands on the *x* axis with power of the frequency bands on the *y* axis during compulsions in ALIC. **c**, Violin plots of PAC strength during compulsions in the highlighted areas in **b**, compared with baseline. Pairwise comparison marks significant difference (one-tailed Wilcoxon signed-rank test). Solid white line, median; transparent white line, quartiles; *P = 0.030. a.u., arbitrary units.

As LFP oscillations reflect temporal coordination of neuronal activity, interaction of these oscillations across frequency bands may play an important role in cognitive processes^26^. Thus, we hypothesized that so-called phase–amplitude coupling (PAC) of subcortical neural oscillations may be disturbed in repetitive, compulsive behavior. To explore this, we calculated the cross-frequency coherence of LFP in basal ganglia and associated white-matter regions during symptom provocation (Fig. 3a). We further explored the striking signal observed in ALIC, and determined that PAC in this region was significantly stronger than semi-randomized PAC during compulsion (*P* = 0.022) but not baseline, obsession or relief states. Three of these ALIC–compulsion cross-frequency interactions (as outlined in Fig. 3b: theta–alpha (5–6 Hz, 9–11 Hz), theta–beta (5–6 Hz, 17–24 Hz) and beta–gamma (13–22 Hz, 30–40 Hz)) were selected for further analysis (note that the prominent 15–30 Hz coupling likely reflects interactions within the beta band itself). Only theta–alpha mean PAC was significantly stronger during compulsions compared with baseline in ALIC (Fig. 3c, one-tailed Wilcoxon signed-rank test, *Z* = –1.89, *P* = 0.030).

## Discussion

Discovering electrophysiological disease markers of OCD is critical for improving outcomes of neuromodulatory interventions such as DBS. Here, we have identified intracranial electrophysiological correlates of several cardinal features of OCD across cortico-striatal network nodes. Crucially, as an essential control lacking from many study designs, in our study we control for the contribution of movement during compulsions, differentiating between brain regions where increased LFP power relates to compulsive feeling from those where it relates to (compulsive) action.

Using the Lead-DBS toolbox^27^ to reconstruct, visualize and localize the DBS leads in each patient individually, combined with additional verification and refinement of electrode locations, we determined that our LFP data were recorded from brain structures whose functions fit in the line of evidence that CSTC-circuit dysfunction is the core pathophysiology of OCD. As a key node in this system, the GPe can be subdivided into (anterior) limbic, (medial) associative and (posterior) sensorimotor functional territories^28,29^. Our recordings target the most anterior, limbic portion of GPe, which is speculated to be of therapeutic relevance for psychiatric disorders, and where disruption of inhibitory neurotransmission is shown to induce stereotypies in primates^30^. Yet, this delineation may be more complex. Thus, for the purpose of this study, we took the additional step of subdividing our GPe recording sites into more anterior (aGPe) and more posterior (pGPe) parts of anterior GPe, and indeed find subtle divergence of electrophysiological signatures in our most anterior and more posterior subdivisions of anterior GPe.

**Extended Data Fig. 1:**
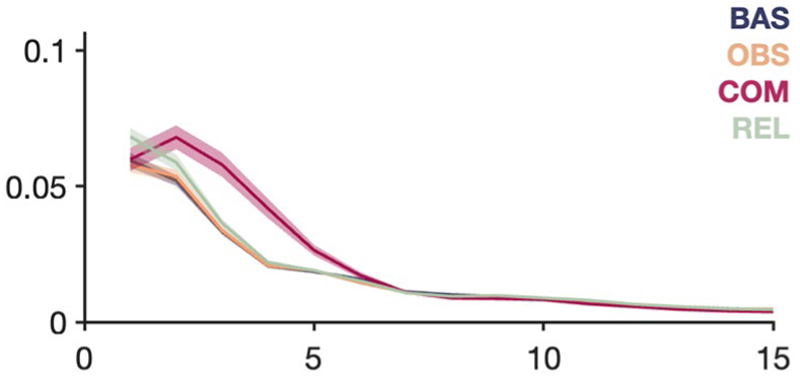
Power spectra (mean±SEM) of LFP recorded during the experimental states in the pooled n=16 electrode pairs that were excluded from analysis as their placement fell wholly or partially across anatomical borders. Compare to Fig. 2b.

Although compulsions increased the power of delta oscillations from baseline across all brain areas targeted in our study (which generalizes to the *n* = 16 electrode pairs that were excluded from analysis due to anatomy; Extended Data Fig. 1), qualitative differences between them imply that this signature was not simply volume-conducted from a single brain region: its spatial distribution was regionally distinct across recording sites, it persisted only in a subset of brain regions during compulsions that were not implicitly linked to physical movement and it correlated with experience of symptom severity only in aGPe. Thus, our data suggest that subcortical delta oscillations are meaningfully different across basal-ganglia structures and that they can reflect compulsivity beyond their purported role in mediating compulsive movements. Alpha-oscillation power similarly increased in all brain areas during compulsions, yet only related to non-motor/mental compulsions in pGPe, and did not correlate with symptom severity. Increased power of theta and beta oscillations during compulsions was region specific, and phase–amplitude interactions were only affected in ALIC. This heterogeneity of our findings across frequency bands and brain regions suggests that we have identified several distinct potential biomarkers that may reflect disparate behavioral aspects of OCD symptomatology.

Understanding of the functional relevance of subcortical oscillatory frequencies in psychiatric disease is limited, as only recent advances have made it possible to record deep-brain LFP in chronically implanted patients. However, human electroencephalography (EEG) studies have linked global, cortical delta activity to motor preparation and transfer of sensory information^31–33^. Delta activity may be particularly suited for this type of communication, as its low-frequency oscillations provide long time windows, allowing larger ensembles of neurons to synchronize their activity, with resulting large oscillation amplitude that may facilitate interaction between distant brain regions. This mechanism may mediate psychiatric symptoms across the CSTC circuit, that is, we speculate that the increased delta power observed in our study reflects compulsive feeling by strongly coupling neuronal activity in basal-ganglia nuclei and cortical areas.

A compelling characterization of OCD posits compulsions as goal-directed actions to mitigate the anxiety induced by obsessions^34^. In this sense, it is unexpected that we did not find obsessions to be represented in deep-brain structures as robustly as compulsions. One interpretation is that obsessions are more strongly represented elsewhere in the brain, and thus, in our study, we identified only the subcortical reflection of this activity. Following an alternative model of OCD, where pathological disinhibition of the prefrontal cortex allows habits to escalate into compulsions, obsessions are interpreted as patients’ post hoc rationalization of their behavior, instead of a driving force^35^. The construct of our study, where obsession and compulsion states were induced separately and in sequence, precludes us from interpreting the relevance of this latter model in our data.

One origin of the LFP signal is synchronization of neuronal post-synaptic potentials by afferent activity^15^; in the OCD-CSTC circuit, increased subcortical LFP power may therefore reflect pathological cortical inputs^36^, which renders the circuit inflexible, resulting in compulsive behavior. Indeed, our team has previously shown that NAc DBS in OCD attenuates symptom-provocation-increased low-frequency frontal EEG power^8^. More recent work in OCD patients has identified connectivity between the NAc and frontal cortex in theta and alpha bands^37^ and shown that bed nucleus of the stria terminalis (BNST)/ALIC DBS reduces theta power but increases the power of higher-frequency oscillations in local LFP and cortical EEG^38^. Finally, the connectivity strength of midfrontal EEG and ventral capsule/ventral striatum delta LFP is found to correlate with OCD symptoms^39^. Less is known about the involvement of GPe, which mediates the ‘indirect’ (net inhibitory) CSTC pathway, conveying neuronal activity between striatum and thalamus. Although OCD symptoms have long been hypothesized to result from overactivation of the ‘direct’ (net excitatory) pathway^40^, pallidal lesions can result in OCD-type behavior^41–44^, and optogenetic inhibition of GPe-projecting striatal neurons reduced compulsive grooming in the Sapap3-knockout mouse model of OCD^45^. Thus, taken together, we hypothesize that the LFP biomarkers identified in our study reflect overactivation of multiple nodes of the CSTC circuit, and that DBS might disrupt the resulting pathological feedback loops, restoring behavioral flexibility.

Interactions of LFP oscillations across frequency bands are believed to underlie information processing^46^. In particular, phase-to-power coupling is hypothesized to facilitate the integration of activity between neuronal networks. Previously, our team has shown that ALIC DBS suppresses cortical PAC in OCD patients^47^, possibly attenuating pathological CSTC hyperconnectivity. In the current study, we did indeed identify increased PAC in ALIC during compulsions. As, canonically, white matter does not generate its own LFP, this signal may be volume-conducted from elsewhere. However, altered cross-frequency coupling was not found in any of the adjacent gray-matter regions examined in this study, which implies that the abnormal ALIC phase–amplitude interactions reflect a unique biomarker of compulsion, distinct from the NAc/CSTC-input and GPe/indirect-pathway biomarkers described above. In fact, it has been suggested that LFP recorded from white matter may reflect distant gray-matter activity traveling through axonal fiber tracts^48^, in which case we believe that increased ALIC PAC reflects pathological coupling of cortical networks in OCD.

Although a number of subcortical areas are commonly targeted with DBS for OCD, these are generally connected via ALIC to the CSTC circuit^5^ and thus typically reduce symptoms equally well^49^ by modulating the same prefrontal cortical regions^50^. Our findings corroborate this to some extent, as we find a compulsion-related, generalized power increase of delta oscillations throughout our basal-ganglia targets. However, the frequency, spatial, behavioral and cross-frequency heterogeneity of our other biomarkers might reflect different aspects of OCD symptomatology and interactions with non-shared neuronal networks. Targeting these novel, heterogeneous biomarkers with DBS might ameliorate separate dimensions of OCD symptomatology such as compulsivity or anxiety, which may be quantified more easily as separate treatment outcomes in individual patients.

In addition, identification of immediate biomarkers of OCD and separate dimensions of response may allow more efficient, temporally specific targeting of symptoms with adaptive DBS. Biomarker-led closed (feedback)-loop DBS as treatment for psychiatric disorders has been the topic of recent (case) studies, which though limited in number of patients and behavioral controls are promising in their results^22,51,52^. The robustly defined, anatomically restricted, group-level neural correlates of OCD we identified across many patients in the current study may substantially aid these efforts, as they enable general, standardized protocols for biomarker detection in patients as a group (circumventing time-consuming individualized approaches), from the very same electrodes that are used to stimulate (thus the translation from detection to intervention may be performed on the same device), paving the way toward biomarker-led closed-loop DBS as treatment for OCD. Moreover, identification of reliable neuronal biomarkers of OCD may also benefit application of other neuromodulatory interventions, such as transcranial magnetic stimulation.

**Extended Data Fig. 2:**
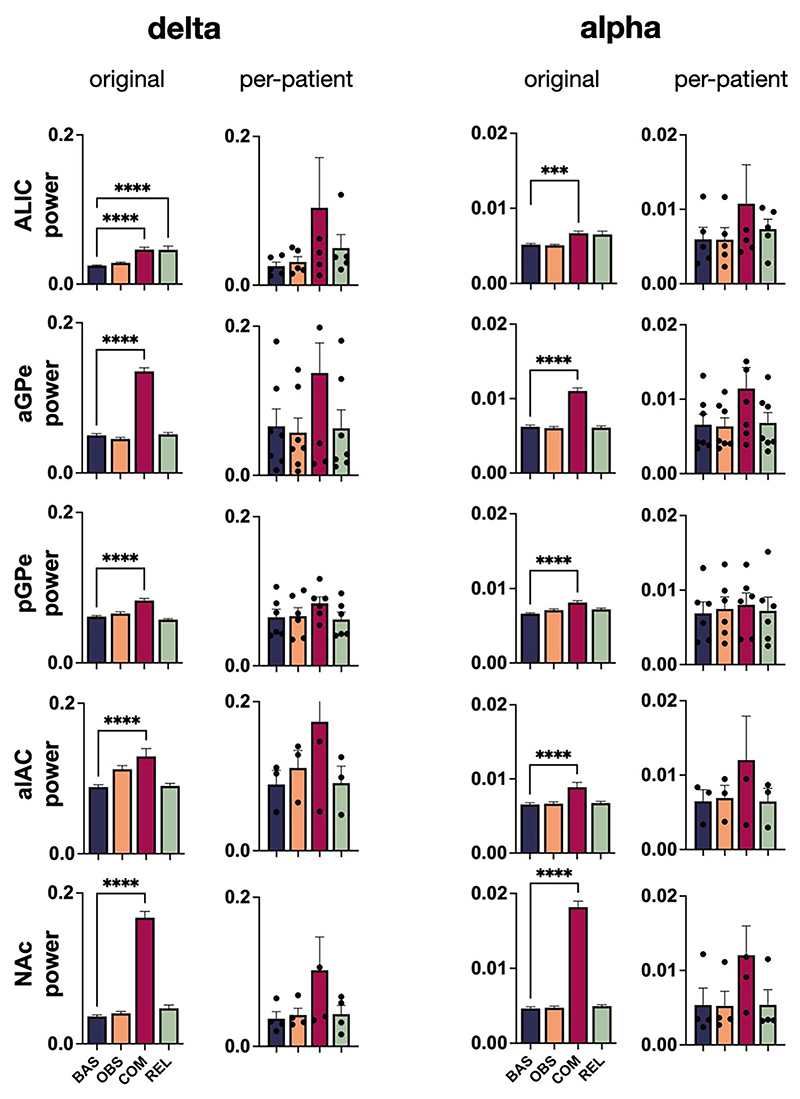
Bar graphs (mean+SEM) of spectral LFP power in delta and alpha frequency bands during the experimental states in each brain region. Left, duplicated from Fig. 2c: pairwise comparisons mark significantly different power during obsession, compulsion, and relief from baseline (Bonferroni-corrected one-way ANOVA with Dunnett’s multiple comparisons test). Right, same data as Left, but plotted on the basis of individual patients. Although the number of patients in each anatomical group is relatively low (ALIC n=5, aGPe n=7, pGPe n=6, alAC n=3, NAc n=4), the results (not analyzed statistically) still follow our original conclusions, that compulsions increase low-frequency LFP power compared with baseline.

Some limitations should be taken into consideration in interpreting our findings. Although we believe ours is the largest cohort of intracranial electrophysiological recordings in OCD to date, the absolute number of patients is still relatively low; therefore, analyses on a perpatient level might have been underpowered (Extended Data Fig. 2), and it was not possible to stratify results by OCD type, comorbidities, medication use and so on. Our behavioral paradigm was designed to subdivide OCD dynamics into four separate states (baseline, obsession, compulsion, relief). Although effective, it is debatable whether this classification provides the best approach to discovering biomarkers for treatment of a disorder that consists of such a broad and dynamic assembly of experience and manifestation between patients.

In fact, immediate effects of DBS typically affect patient mood, anxiety and confidence before alleviating obsessions and compulsions, and it is therefore possible that, despite our characterization of the behavior states in our paradigm as ‘obsessive’ or ‘compulsive’, the neural correlates we find reflect more subtle representations thereof. For instance, our correlation of delta power with obsession severity is similar to the reported low-frequency power increase immediately preceding loss-of-control eating^53^, and our corresponding delta-power and anxiety decrease during compulsions is similar to the reported delta-power decrease with OCD symptom intensity^22^; these findings might be different classifications of the same neural signal. Moreover, as Fig. 2e illustrates, neural signals during compulsions may relate to more than just the compulsions themselves, reflecting reduction in VAS anxiety. In the same vein, it is possible that our choice of VAS to monitor throughout symptom provocation does not accurately reflect patients’ experience of their symptomatology; for instance, basal-ganglia LFP might correlate more strongly with feelings of distress or disgust elicited during symptom provocation, which were not evaluated in our study.

Furthermore, we chose to restrict our analyses to recordings taken from electrode pairs localized within CSTC structures, thus defining our results on the basis of current understanding of presupposed networks of OCD. It is, however, possible that LFP recorded differentially from a combination of brain regions could reveal other signatures of OCD dynamics not identified in our study. Additionally, as an exploratory study, our results must be replicated in a hypothesis-driven manner to verify how these biomarkers reflect OCD symptoms, and whether they can be used successfully as triggers for closed-loop DBS or other neuromodulatory intervention. Finally, although we controlled for the effects of movement on neuronal correlates of compulsion by separately analyzing patients on the basis of their motor activation, future experiments would benefit from a more comprehensive characterization of movement effects on basal-ganglia LFP by controlling for non-compulsive movement-related neuronal activity.

Ultimately, we describe here an approach to identifying intracranial biomarkers of psychiatric disease using an unprecedentedly large group of patients that is innovative in multiple aspects: the data were recorded from electrodes that can be directly implemented for treatment, the behavioral paradigm was individually tailored to each patient and we differentiated our data between multiple brain areas due to highly refined anatomical localization of our recording sites. With this paradigm we found novel, group-level neural signatures of OCD obtained from sensing DBS recordings, which may prove invaluable in the pursuit of biomarker-targeted neuromodulatory intervention.

## Methods

### Participants

Between June 2015 and March 2020, 11 patients with treatment-resistant OCD indicated for DBS were recruited at the outpatient clinic of the psychiatry department of the Amsterdam UMC, location AMC. The study was approved by the Medical Ethics Committee of the Academic Medical Center, University of Amsterdam, and registered in the Netherlands Trial Register under trial number NL7486, https://onderzoekmetmensen.nl/en/trial/50295. Before participation, all patients provided written informed consent.

Participants included in the study met the following inclusion criteria: primary diagnosis of OCD according to DSM and indication for DBS implantation. This patient group was defined as treatment resistant with insufficient response to at least two treatments with a selective serotonin reuptake inhibitor at maximum dosage for >12 weeks, one treatment with clomipramine at maximum dosage for >12 weeks and at least one augmentation trial with an atypical antipsychotic and a selective serotonin reuptake inhibitor for 8 weeks. In addition, patients must have undergone one cognitive behavioral therapy trial at an OCD expert center, involving individual sessions, group day treatment or admission^2^. Patients were included following a new indication for DBS (ten patients) or an indication for stimulator replacement after successful DBS (one patient). Patients were excluded from the study in the case of substance or alcohol abuse 6 months before participation.

### DBS-implantation surgery

Implantation of the DBS system was performed under general anesthesia. Frame-based magnetic resonance imaging (MRI) was used for target determination. A detailed description of the surgical procedure was previously reported^2^. In short, Medtronic model 3389 leads (used off-label) with four contact electrodes (each 1.5 mm in length, spaced 0.5 mm apart, with 7.5 mm total spread) were implanted bilaterally in the brain, following the ALIC, with the most ventral contact targeting the NAc (Fig. 1c). The leads were connected via subcutaneous Medtronic extension cables to an Activa PC + S sensing neurostimulator (Medtronic model 37604, used off-label), which was placed in the right infraclavicular pocket. This telemetry device is able to both stimulate and record LFP at its contact electrodes. Patients received standard medical care after the procedure.

### Study design

The experiment was conducted in the clinic once patients had recovered from surgery, which was two to three weeks after device implantation, before the DBS system being activated. In two exceptional cases, recordings were done after DBS had already been activated; stimulation was temporarily turned off (an hour beforehand) for these recordings.

Recordings were done during a symptom-provocation task in which patients experienced and performed, respectively, their patient-specific obsessions and compulsions (Fig. 1a, as previously reported^25^): 3 min of LFP was recorded during four behavioral states (baseline, obsession, compulsion, relief). Appropriate personalized symptom-provocation methods were selected in consultation with the patient before the experiment.

During the 3-min baseline recordings, patients were asked to sit still and watch a neutral movie. This was followed by personalized obsession provocation (for example, touching the floor in case of contamination fear), after which patients were asked to sit still and experience their obsessions for another 3-min LFP recording. Subsequently, patients were allowed to perform their compulsions (for example, washing their hands in case of contamination fear) while LFP was recorded for 3 min or longer, until their urge resolved. Finally, patients were asked to sit still and experience their relief for another 3-min LFP recording. All but one patient underwent four iterations of the entire protocol.

### Outcome measures

Neural activity was recorded using the 8180 Sensing Programmer SW (Medtronic) in montage mode, obtaining LFP differentially between two electrodes for 30 s each in succession, across all six electrode combinations on the lead, simultaneously in both hemispheres (sampling rate 422 Hz, gain 2,000, bandpass filtered 0.5–100 Hz).

Severity of obsession, compulsion, agitation, anxiety, depressive mood and avoidance was monitored throughout the experiment at the beginning and end of each behavioral state using a VAS, on which patients rated each from 1 to 10 (where higher scores imply more severe symptoms, Fig. 1a).

The Y-BOCS^54^ was used to measure the severity of OCD symptoms pre-surgery, providing insight into the clinical status of the participants during the experiment (scores are divided into obsessive and compulsive subscores, with a total score range from 0 to 40, where higher scores imply more severe symptoms). Demographic, clinical and experimental details of the study group are summarized in Table 1 (as previously reported^25^). Patient age range was 32–68 years (mean 49, s.d. 10.9).

### Anatomical localization

The position of the implanted DBS leads was verified using the Lead-DBS toolbox v.2.5.2^27^ in MATLAB R2021a (MathWorks). In short, post-operative stereotactic computerized tomography was merged with preoperative *T*_1_ MRI using a two-stage linear registration (rigid followed by affine) as implemented in Advanced Normalization Tools^55^ for each patient individually. Coregistered scans were inspected, refined, corrected for postoperative brain shift as needed and subsequently normalized into ICBM 2009b nonlinear asymmetric MNI space^56^ using the SyN-registration approach as implemented in Advanced Normalization Tools. DBS electrodes were automatically pre-reconstructed in native and template space using the PaCER algorithm^57^, visually inspected and manually refined as needed. Anatomical localization of each pair of adjacent electrodes was determined using an adaptation of the DISTAL atlas^58^ in Lead-DBS, after which results were visually verified to individual patient computerized tomography–MRI coregistered electrode trajectories, and refined using two additional atlases^59,60^. The Lead-Group toolbox v.2.5.2^61^ was used to visualize recording sites (Fig. 1c,d) and the distribution of baseline-subtracted delta power onto them (Fig. 2f), using an adaptation of the DISTAL atlas^58^.

### Data analysis

Spectral analyses of LFP were performed in MATLAB R2022b (Math-Works) using FieldTrip^62^ and custom-made routines. Only data recorded from adjacent electrode pairs that were unambiguously localized within a clearly defined anatomical region were used (50 out of a total 66 pairs). Due to amplifier artifacts, the first 1,210 samples were removed from each recording, leaving 27 s of continuously recorded LFP for further analysis. Power-line artifacts were removed using a discrete Fourier transform filter at 50 Hz and its second and third harmonics, and an additional band-stop filter at 47–53 Hz. Data were segmented into 1-s epochs, which were visually inspected, manually artifact-rejected, *Z*-scored (to equalize the contribution of different electrodes across patients), demeaned, Hanning-tapered and Fourier-transformed. Spectral estimates were evaluated for significant differences from baseline at *P* < 0.05 (Fig. 2b), using a non-parametric randomization test, corrected for multiple comparisons, for each brain region across all frequency bins (as previously reported^18^). Frequency bands were defined as delta 1–3 Hz, theta 4–7 Hz, alpha 8–12 Hz, beta 13–29 Hz, gamma 30–80 Hz.

PAC analyses (Fig. 3a) were performed on the basis of the cross-frequency coherence between a low-frequency oscillation and the power envelope of a high-frequency oscillation^63^. The statistical significance of PAC was assessed by 100 repetitions of randomly shifting the signal >3,000 data points with regards to the power and recalculating their coherence; the proportion of maximum randomly shifted coherence values obtained that lie above the original coherence value determine the *P* value.

Additional statistical analyses were Bonferroni-corrected one-way ANOVA with Dunnett’s multiple comparisons, Bonferroni-corrected unpaired *t*-test, Pearson correlation, linear regression and one-tailed Wilcoxon signed-rank test. These analyses were performed using Prism 9.1.2 (GraphPad) and custom-made routines in MATLAB R2022b. The significance level was set at *P* < 0.05.

### Reporting summary

Further information on research design is available in the Nature Portfolio Reporting Summary linked to this article.

## Extended Data

**Extended Data Table 1:** Number of samples for each frequency bin in Fig. 2b

FIGURE 2B NUMBER OF SAMPLES				
Brain region	Baseline	Obsession Compulsion		Relief
ALIC	1004	1108	969	996
aGPe	1255	1291	1702	1191
pGPe	1783	1769	1569	1690
alAC	628	675	487	575
NAc	491	512	775	491

**Extended Data Table 2:** Statistics for Fig. 2c Bonferroni-corrected one-way ANOVA with Dunnett’s multiple comparisons

FIGURE 2C STATISTICS				
Brain reqion	Frequency band	Anova	Dunnett’s multiple comparison	Effect size
ALIC	*δ*	F (3, 4073) = 14.63, p < 0.0001	BAS vs COM 95% Cl -0.03153 to -0.01155, p < 0.0001 BAS vs REL 95% Cl -0.03105 to -0.01121, p < 0.0001	0.7622 0.7477
	*θ*	F (3, 4073) = 21.91, p< 0.0001	BAS vs COM 95% Cl -0.01169 to -0.005739, p < 0.0001 BAS vs REL 95% Cl -0.007616 to -0.001703, p = 0.0006	0.9924 0.5302
	*α*	F (3, 4073) = 9.874, p < 0.0001	BAS vs COM 95% Cl -0.002435 to -0.0005882, p = 0.0004	0.3120
aGPe	*δ*	F (3, 5435) = 173.9, p < 0.0001	BAS vs COM 95% Cl -0.09640 to -0.07381, p < 0.0001	1.0251
	*θ*	F (3, 5435) = 210.6, p < 0.0001	BAS vs COM 95% Cl -0.03243 to -0.02535, p < 0.0001	1.4155
	*α*	F (3, 5435) = 67.26, p < 0.0001	BAS vs COM 95% Cl -0.005833 to -0.003772, p < 0.0001	0.5618
	*β*	F (3, 5435) = 14.08, p< 0.0001	BAS vs COM 95% Cl -0.0007131 to -0.0002247, p < 0.0001	0.1678
pGPe	*δ*	F (3, 6807) = 28.66, p < 0.0001	BAS vs COM 95% Cl -0.02838 to -0.01464, p < 0.0001	0.3474
	*θ*	F (3, 6807) = 32.74, p < 0.0001	BAS vs COM 95% Cl -0.009770 to -0.005884, p < 0.0001	0.5578
	*α*	F (3, 6807) = 11.35, p< 0.0001	BAS vs COM 95% Cl -0.002125 to -0.0008903, p < 0.0001	0.2502
	*β*	F (3, 6807) = 11.42, p < 0.0001	BAS vs COM 95% Cl -0.0005296 to -0.0002128, p < 0.0001	0.2125
alAC	*δ*	F (3, 2361) = 11.38, p< 0.0001	BAS vs COM 95% Cl -0.06004 to -0.02164, p < 0.0001	0.5392
	*α*	F (3, 2361) = 8.800, p < 0.0001	BAS vs COM 95% Cl -0.003583 to -0.001109, p < 0.0001	0.3844
NAc	*δ*	F (3, 2265) = 123.6, p < 0.0001	BAS vs COM 95% Cl -0.1510 to -0.1110, p < 0.0001	2.6630
	*θ*	F (3, 2265) = 197.0, p < 0.0001	BAS vs COM 95% Cl -0.04901 to -0.03810, p < 0.0001	2.6787
	*α*	F (3, 2265) = 166.8, p < 0.0001	BAS vs COM 95% Cl -0.01536 to -0.01170, p < 0.0001	2.6553
	*β*	F (3, 2265) = 151.0, p< 0.0001	BAS vs COM 95% Cl -0.005192 to -0.003908, p < 0.0001	3.7418
	*γ*	F (3, 2265) = 83.73, p < 0.0001	BAS vs COM 95% Cl -0.0006855 to -0.0004726, p < 0.0001	1.7942

**Extended Data Table 3:** Statistics for Fig. 2d delta Bonferroni-corrected unpaired t-test

FIGURE 2D STATISTICS *δ*				
Brain region	Baseline vs Motor	Effect size	Baseline vs Non-motor	Effect size
ALIC	t(1432) = 8.184, 95% Cl 0.01375 to 0.02243, p < 0.0001	0.6440	t(537) = 3.017, 95% Cl 0.01051 to 0.04976, p < 0.0027	1.0465
aGPe	t(2298) = 13.83, 95% Cl 0.08408 to 0.1119, p < 0.0001	1.1615	t(655) = 3.632, 95% Cl 0.01614 to 0.05413, p < 0.0003	0.4462
pGPe	t(1944) = 5.321,95% Cl 0.01167 to 0.02530, p < 0.0001	0.2764	t(1404) = 4.801,95% Cl 0.01561 to 0.03718, p < 0.0001	0.4749
alAC	t(1113) = 4.186, 95% Cl 0.02170 to 0.05998, p < 0.0001	0.5392	-	-
NAc	t(1052) = 12.44, 95% Cl 0.1306 to 0.1795, p < 0.0001	2.9403	t(210) = 0.3585, 95% Cl -0.008835 to 0.01276, p = 0.7203	0.0687

**Extended Data Table 4:** Statistics for Fig. 2d alpha Bonferroni-corrected unpaired t-test

FIGURE 2D STATISTICS *α*				
Brain region	Baseline vs Motor	Effect size	Baseline vs Non-motor	Effect size
ALIC	t(1432) = 7.573, 95% Cl 0.001583 to 0.002690, p < 0.0001	0.5599	t(537) = 0.2456, 95% Cl -0.001994 to 0.001551, p = 0.8061	-0.0327
aGPe	t(2298) = 10.99, 95% Cl 0.004991 to 0.007159, p < 0.0001	1.6515	t(655) = 1.216, 95% Cl -0.001009 to 0.004290, p = 0.2246	0.1128
pGPe	t(1944) = 3.802, 95% Cl 0.0006399 to 0.002004, p = 0.0001	0.2039	t(1404) = 4.174, 95% Cl 0.001020 to 0.002828, p < 0.0001	0.3533
alAC	t(1113) = 3.634, 95% Cl 0.001069 to 0.003578, p = 0.0003	0.3844	-	-
NAc	t(1052) = 13.79, 95% Cl 0.01408 to 0.01875, p < 0.0001	6.3715	t(210) = 0.4103, 95% Cl -0.002212 to 0.001450, p = 0.6820	-0.0582

## Figures and Tables

**Table 1:** Patient demographics and experiment details

Demographics				During experiment		
Gender	Handedness	Y-BOCS score		OCD subtype	Obsessions induced by	Compulsion behavior
F	Left	8 + 7^a^		Contamination	Touching the floor	Washing hands
F	Right	18 + 18		Religious	Reading a religious text	Praying^b^
F	Right	18 + 17		Contamination/checking	Touching the floor	Washing hands
F	Right	19 + 18		Contamination/checking	Others touching a personal object brought from home	Inspecting^c^ the object
F	Right	19 + 20		Contamination/symmetry	Touching the floor	Washing hands
F	Right	17 + 19		Harm/checking	Thinking about a fire risk	Checking whether there is a fire
F	Right	17 + 16		Contamination/symmetry	Touching the floor	Washing hands
F	Right	17 + 16		Contamination	Touching the door knob/light switch	Washing hands
F	Right	16 + 19		Harm/contamination	Thinking about a personal contaminated object	Checking photographs to see the object is clean
M	Right	11 + 11		Religious	Reading a religious text	Reading a reassuring text
F	Right	17 + 16		Contamination	Touching the floor	Washing hands

F, female; M, male. Yale–Brown Obsessive Compulsive Scale (Y-BOCS) scores are displayed as obsessions + compulsions. ^a^This patient had already undergone DBS treatment and was recruited into the current study after a replacement of the device battery. The original clinical Y-BOCS score was 38. ^b^This patient occasionally wrote. ^c^This patient occasionally washed the object.

## Data Availability

The data that support the findings of this study are not openly available to protect patient privacy. Anatomical localization of electrodes was determined using the DISTAL atlas implemented in Lead-DBS toolbox v.2.5.2 MATLAB R2021a.
